# Replication and characterization of *CADM2* and *MSRA* genes on human behavior

**DOI:** 10.1016/j.heliyon.2017.e00349

**Published:** 2017-07-26

**Authors:** Brian Boutwell, David Hinds, the 23andMe Research Team, Michelle Agee, Babak Alipanahi, Adam Auton, Robert K. Bell, Katarzyna Bryc, Sarah L. Elson, Pierre Fontanillas, Nicholas A. Furlotte, David A. Hinds, Bethann S. Hromatka, Karen E. Huber, Aaron Kleinman, Nadia K. Litterman, Matthew H. McIntyre, Joanna L. Mountain, Carrie A.M. Northover, J.Fah Sathirapongsasuti, Olga V. Sazonova, Janie F. Shelton, Suyash Shringarpure, Chao Tian, Joyce Y. Tung, Vladimir Vacic, Catherine H. Wilson, Jorim Tielbeek, Ken K. Ong, Felix R. Day, John R.B. Perry

**Affiliations:** a School of Social Work, College for Public Health and Social Justice, Saint Louis University, 3550 Lindell Blvd. St. Louis, MO 63103, USA; b Department of Epidemiology, College for Public Health and Social Justice, Salus Center 3545 Lafayette Avenue St. Louis, MO 63104, USA; c 23andMe Inc., 899 W. Evelyn Avenue, Mountain View, California 94041, USA; d Department of Child and Adolescent Psychiatry, VU University Medical Center Amsterdam, Duivendrecht, The Netherlands; e MRC Epidemiology Unit, University of Cambridge School of Clinical Medicine, Box 285 Institute of Metabolic Science, Cambridge Biomedical Campus, Cambridge, CB2 0QQ, UK

**Keywords:** Genetics, Clinical psychology, Psychiatry, Neuroscience, Psychology

## Abstract

Progress identifying the genetic determinants of personality has historically been slow, with candidate gene studies and small-scale genome-wide association studies yielding few reproducible results. In the UK Biobank study, genetic variants in *CADM2* and *MSRA* were recently shown to influence risk taking behavior and irritability respectively, representing some of the first genomic loci to be associated with aspects of personality. We extend this observation by performing a personality “phenome-scan” across 16 traits in up to 140,487 participants from 23andMe for these two genes. Genome-wide heritability estimates for these traits ranged from 5–19%, with both *CADM2* and *MSRA* demonstrating significant effects on multiple personality types. These associations covered all aspects of the big five personality domains, including specific facet traits such as compliance, altruism, anxiety and activity/energy. This study both confirms and extends the original observations, highlighting the role of genetics in aspects of mental health and behavior.

## 1 Introduction

Progress identifying the individual genes underpinning the heritable component (h^2^ = 40–60%) [1] of psychiatric disease and personality has lagged behind that of other complex traits [2]. A number of reasons might explain this lag, including strong evolutionary selection against common alleles with large effects, a lack of large studies with detailed and harmonized trait information, disease misclassification and subjective clinical criteria. Candidate gene studies have yielded few, if any, credible associations that are replicated [2] [3]. In the last few years, suitably powered genome-wide association study (GWAS) meta-analyses have begun identifying handfuls of genetic variants associated with severe psychiatric disease [4] [5]. This success, alongside the arrival of large studies such as UK Biobank [6], prompted the hunt for alleles associated with other behavioral and psychological outcomes. Notably, a recent GWAS on educational attainment [7] identified 74 robustly associated genomic loci, which were subsequently demonstrated to have a causal effect on longevity [8]. Two studies recently reported the first loci convincingly associated with aspects of personality, performing large-scale GWAS in up to ∼300,000 individuals for subjective well-being [9], neuroticism [9], depressive symptoms [9], irritability [10] and risk taking [10]. Amongst these loci, *CADM2* and *MSRA* were initially linked with reproductive onset (age at first sex and birth), with subsequent analyses suggesting the mechanism of effect might act through broader personality domains (risk taking and irritability) [10]. The range of personality phenotypes in this original study was however limited and no replication datasets were available at the time. Our present study builds on this initial observation by replicating and extending these prior associations at *CADM2* and *MSRA*, examining 16 personality constructs using independent data from 23andMe, Inc., a personal genetics company.

## 2 Methods

### 2.1 Human subjects

All 23andMe research participants included in the analysis provided informed consent and answered surveys online according to a human subjects protocol approved by Ethical & Independent Review Services, a private institutional review board.

### 2.2 Personality inventories

Soto and John [11] proposed 10 facet scales based on global Big Five Inventory (BFI) items (i.e., two facet scales for each domain captured in the BFI). We computed the 10 facet scales, as well as the traditional BFI categories, using data collected from a web-based version of the Big Five Inventory. Separately, we used a single question to assess risk comfort (see supplementary note for additional information). Items were coded such that higher scores on each dimension corresponded to the increased presence of a particular trait (i.e., higher scores on the “compliance” items represent more compliant and agreeable tendencies). All phenotypes were coded on a 5-point scale according to the answers given to the personality questions listed in the Supplementary note. For phenotypes based on more than one question, an individual's score is the average of their answers across questions.

### 2.3 Genetic analysis

Genotyping of research participants was performed as previously described [12]. Genotypes for rs1865251 and rs658385 were imputed (minimum imputation quality > 0.95) using the March 2012 “v3” release of 1000 Genomes reference haplotype panel. Genetic association results were obtained from linear regression models assuming additive allelic effects. These models included as covariates − age, sex and the top five genetically determined principal components to account for population structure. The reported SNP association test P-values were computed from likelihood ratio tests. As the aim of this study is to replicate the original observations and explore the breadth of effect, we accepted a nominal level of statistical significance (P < 0.05) as evidence for association. We have also noted associations that pass the most conservative multiple-testing threshold of P < 0.002 (0.05/(16*2)).

Genome-wide SNP-based heritability was estimated using LD score regression, implemented in the LDSC software package. Default software parameters were used and only common SNPs (MAF > 5%) present in HapMap 3 were considered.

## 3 Results

**Fig. 1:**
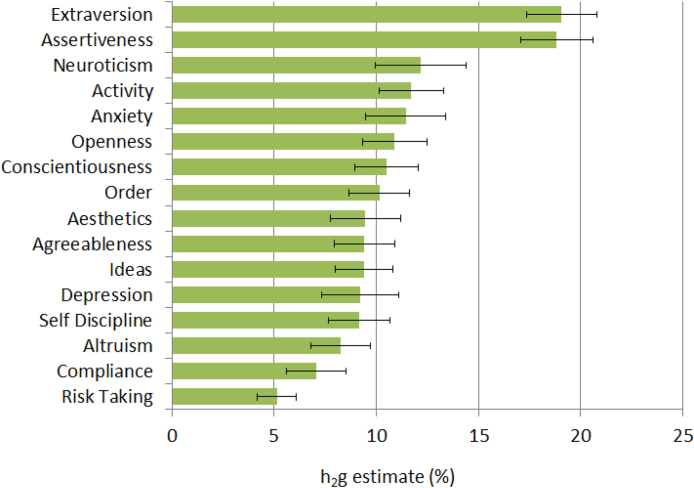
Genome-wide SNP-based heritability estimates for the 16 personality traits tested.

Phenotypes and genotypes were available in up to 140,487 research participants from 23andMe, independent from the previous study. A total of 16 personality constructs were defined on the basis of answers from 45 questions (Supplementary Table 1). We estimated a significant SNP-based heritability for each of these 16 traits (Fig. 1), ranging from 5.1% (risk taking) to 19.1% (extraversion).

**Fig. 2:**
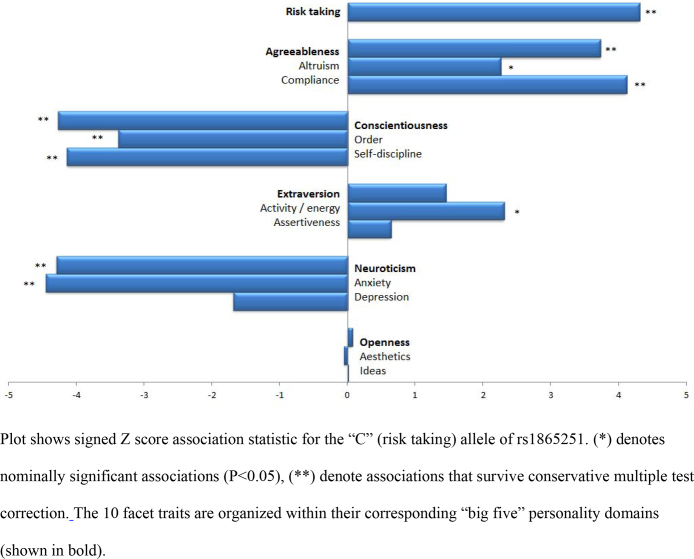
Personality types associated with the *CADM2* risk taking allele.

The *CADM2* risk taking allele (best Hapmap2 proxy = rs1865251, “C” allele) was significantly associated (P = 1.6 × 10^−5^) with outcome to the question “Overall, do you feel comfortable or uncomfortable taking risks?”, confirming the original observation. An additional nine personality traits exhibited a nominal (P < 0.05) association (Fig. 2), the most significant of which was reduced anxiety levels (P = 8.6 × 10^−6^).

**Fig. 3:**
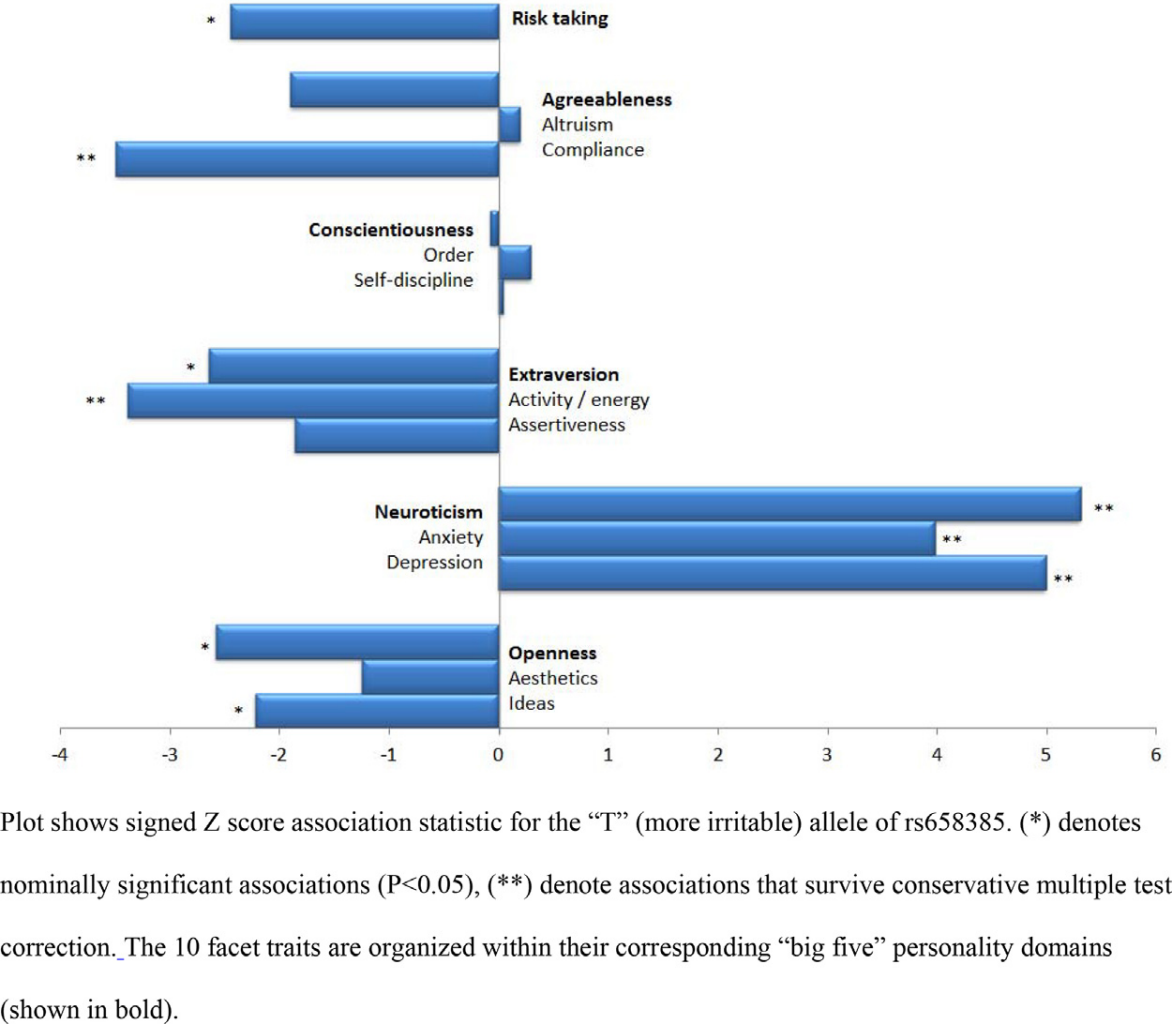
Personality types associated with the *MSRA* increased irritability allele.

For *MSRA*, there was no single personality trait that matched self-reported irritability in the 23andMe dataset; however, the allele previously associated with increased irritability was significantly associated (Fig. 3) with reduced energy and enthusiasm (“activity”, P = 7.2 × 10^−4^), less compliance (P = 4.8 × 10^−4^), more depressive feelings (P = 6 × 10^−7^), more neuroticism (P = 1.1 × 10^−7^), less creative thinking (“ideas”, P = 0.02), more introversion (P = 8 × 10^−3^), more anxiousness (P = 6.9 × 10^−5^), less openness to new ideas (P = 9.8 × 10^−3^) and more adverse feelings to taking risks (P = 0.01).

## 4 Discussion

Large-scale GWAS meta-analyses are now routinely comprised of hundreds of thousands of samples, and as a result possess the requisite statistical power for elucidating the genetic basis of complex human personality traits. Within the last year, several studies have identified the first robustly associated genetic variants associated with these outcomes, with a handful of loci discovered for subjective well-being [9], neuroticism [9, 13], extraversion [13] and conscientiousness [13]. In addition, we previously reported genetic variation in *MSRA* and *CADM2* linked to a range of behavioral reproductive outcomes [10]. Here, the signal in *CADM2* was initially linked to a single self-reported behavioral outcome − risk taking propensity − in the UK Biobank study. The risk taking allele was associated with earlier age at first sex, increased number of sexual partners, a higher number of offspring, increased body mass index and decreased cognitive processing speed. Our current study replicates and extends this observation, demonstrating that this risk taking behavior increasing allele is broadly associated with a “care free” and optimistic personality type. *CADM2* is widely expressed in a number of neuronal tissues and implicated in glutamate signaling, γ-aminobutyric acid transport and neuron cell–cell adhesion [14]. In contrast, the *MSRA* allele promoting later age at first sex was first demonstrated to have an association with increased self-reported irritability. We extend this observation to portray a personality type dominated by anxiety, neuroticism, depression, lack of compliance and reduced energy levels. Since our initial observation this locus has been reported at genome-wide significance for neuroticism [9, 13], however our current result demonstrates a broader personality influence.

We note that in several instances, the phenotypic direction of effect at each locus aligns with the phenotypic correlations observed for the subscales in prior work [11]. For instance, the *MSRA* allele linked to increased neuroticism (anxiety and depression) also decreased levels of extraversion broadly (and activity/energy, more narrowly) which are negatively correlated at the phenotypic level. In contrast, *CADM2* appeared to be associated with increased aspects of agreeableness and decreased levels of conscientiousness, which display a positive phenotypic correlation. These findings underpin the biological complexity and pleiotropy that can exist at an individual locus. Our study has several limitations, notably that we are yet to identify the functional variants or genes responsible for the association signals. Further experimental work will be required to identify the likely effector gene behind this association, particularly at the *MSRA* locus which has extensive linkage disequilibrium. The large sample sizes used here and modest statistical significance observed also reinforce the challenges that lie ahead in more fully elucidating the genetic architecture and molecular mechanisms underpinning personality. With this in mind, what is clear is that only international collaboration and GWAS meta-analyses comprised of millions of individuals, ideally with harmonized phenotypes, will be required to make substantial progress in this area. Capturing complex personality types using simple summarized variables is also an inherently limited approach, however there will always be a trade-off between phenotypic precision and sample size.

In summary, our study and others demonstrates that genetic studies are now suitably powered to identify reproducibly associated genetic determinants of personality. Genetic correlation analyses have previously demonstrated significant overlap in the genes responsible for “normal” personality variation and those involved in severe psychiatric disease [13]. This suggests that expanded personality studies may eventually provide complementary insight into the etiology of severe disease states, when a more substantial fraction of the heritability of personality type is established. It also paves the way for the understanding of causes and consequences of these conditions through the use of Mendelian randomization and similar methods.

## Declarations

### Author contribution statement

Brian Boutwell, Jorim Tielbeek, Ken K. Ong, Felix R. Day: Conceived and designed the experiments; Wrote the paper.

David Hinds, John R.B. Perry: Conceived and designed the experiments; Performed the experiments; Analyzed and interpreted the data; Wrote the paper.

The 23andMe Research Team: Performed the experiments; Analyzed and interpreted the data; Contributed reagents, materials, analysis tools or data.

### Funding statement

This work was supported by the Medical Research Council [Unit Programme number MC_UU_12015/2].

### Competing interest statement

The authors declare no conflict of interest.

### Additional information

The 23andMe Research team consists of: Michelle Agee, Babak Alipanahi, Adam Auton, Robert K. Bell, Katarzyna Bryc, Sarah L. Elson, Pierre Fontanillas, Nicholas A. Furlotte, David A. Hinds, Bethann S. Hromatka, Karen E. Huber, Aaron Kleinman, Nadia K. Litterman, Matthew H. McIntyre, Joanna L. Mountain, Carrie A.M. Northover, J. Fah Sathirapongsasuti, Olga V. Sazonova, Janie F. Shelton, Suyash Shringarpure, Chao Tian, Joyce Y. Tung, Vladimir Vacic, Catherine H. Wilson. The 23andMe Research team are based at: 23andMe Inc., 899 W. Evelyn Avenue, Mountain View, California 94041.

## Appendix A Supplementary data

Supplementary Note
