# Supplementary material for: Replication and characterization of CADM2 and MSRA genes on human behavior
Source: Heliyon. 2017 Jul 26;3(7):e00349. doi: 10.1016/j.heliyon.2017.e00349 (PMC5537199; doi:10.1016/j.heliyon.2017.e00349)
Supplement: Supplementary Note [file mmc1.docx]

**Supplementary Note**

23andMe, Inc. participants were asked the following questions, with the following responses categorically graded 1-5: “Strongly disagree,” “Disagree a little,” “Neither agree nor disagree,” “Agree a little,” and “Strongly agree”:

(1) I am someone who *is talkative*.

(2) I am someone who *tends to find fault with others*.

(3) I am someone who *does a thorough job*.

(4) I am someone who *is depressed, often feels sad*.

(5) I am someone who *is original, comes up with new ideas*.

(6) I am someone who *is reserved*.

(7) I am someone who *is helpful and unselfish with others*.

(8) I am someone who *can be somewhat careless*.

(9) I am someone who *is relaxed, handles stress well*.

(10) I am someone who *is curious about many different things*.

(11) I am someone who *is full of energy*.

(12) I am someone who *starts quarrels with others*.

(13) I am someone who *is a reliable worker*.

(14) I am someone who *can be tense*.

(15) I am someone who *is ingenious, a deep thinker*.

(16) I am someone who *generates a lot of enthusiasm*.

(17) I am someone who *has a forgiving nature*.

(18) I am someone who *tends to be disorganized*.

(19) I am someone who *worries a lot*.

(20) I am someone who *has an active imagination*.

(21) I am someone who *tends to be quiet*.

(22) I am someone who *is generally trusting*.

(23) I am someone who *tends to be lazy*.

(24) I am someone who *is emotionally stable, not easily upset*.

(25) I am someone who *is inventive*.

(26) I am someone who *has an assertive personality*.

(27) I am someone who *can be cold and aloof*.

(28) I am someone who *perseveres until the task is finished*.

(29) I am someone who *can be moody*.

(30) I am someone who *values artistic, aesthetic experiences*.

(31) I am someone who *is sometimes shy, inhibited*.

(32) I am someone who *is considerate and kind to almost everyone*.

(33) I am someone who *does things efficiently*.

(34) I am someone who *remains calm in tense situations*.

(35) I am someone who *prefers work that is routine*.

(36) I am someone who *is outgoing, sociable*.

(37) I am someone who *is sometimes rude to others*.

(38) I am someone who *makes plans and follows through with them*.

(39) I am someone who *gets nervous easily*.

(40) I am someone who *likes to reflect, play with ideas*.

(41) I am someone who *has few artistic interests*.

(42) I am someone who *likes to cooperate with others*.

(43) I am someone who *is easily distracted*.

(44) I am someone who *appreciates art, music, or literature*.

(45) In general, people often face risks when making financial, career, or other life decisions. Overall, do you feel comfortable or uncomfortable taking risks?

Question 45 has the response options “Very comfortable taking risks,” “Somewhat comfortable taking risks,” “Neither comfortable nor uncomfortable taking risks,” “Somewhat uncomfortable taking risks,” and “Very uncomfortable taking risks”, also graded 1 to 5.

Questions 1-44 were condensed down into the following 15 personality scales or facets, based on definitions as described in Soto, C.J. and John O.P., Journal of Research in Personality (2009) (<http://www.colby.edu/visitors/wp-content/uploads/sites/50/2013/08/Soto_John_2009a.pdf>):

“activity,” will be defined with questions 11 and 16.

“aesthetics,” will be defined with questions 30, 41R, and 44.

“agreeableness,” will be defined with questions 2, 7, 12, 17, 22, 27, 32, 37, and 42.

“altruism,” will be defined with questions 7, 22, 27R, and 32.

“anxiety,” will be defined with questions 9R, 19, 34R, and 39.

“assertiveness,” will be defined with questions 1, 6R, 21R, 26, and 31R.

“compliance,” will be defined with questions 2R, 12R, and 17.

“conscientiousness,” will be defined with questions 3, 8, 13, 18, 23, 28, 33, 38, and 43.

“depression,” will be defined with questions 4 and 29.

“extraversion,” will be defined with questions 1, 6, 11, 16, 21, 26, 31, and 36.

“ideas,” will be defined with questions 10, 15, 25, 35R, and 40.

“neuroticism,” will be defined with questions 4, 9, 14, 19, 24, 29, 34, and 39.

“openness,” will be defined with questions 5, 10, 15, 20, 25, 30, 35, 40, 41, and 44.

“order,” will be defined with questions 8R and 18R.

“self discipline,” will be defined with questions 13, 23R, 28, 38, and 43R.

Separately, “risk comfort” was defined by question 45.
