# Supplementary material for: Replication and characterization of CADM2 and MSRA genes on human behavior
Source: Heliyon. 2017 Jul 26;3(7):e00349. doi: 10.1016/j.heliyon.2017.e00349 (PMC5537199; doi:10.1016/j.heliyon.2017.e00349)
Supplement: Supplementary Table 1 [file mmc2.docx]

**Supplementary Table 1** |Association test statistics for *CADM2* and *MSRA*

| Gene | rsid | Other | Effect | phenotype | pvalue | beta | stderr | Z | N |
| --- | --- | --- | --- | --- | --- | --- | --- | --- | --- |
| *CADM2* | rs1865251 | A | C | activity | 2.09E-02 | 0.012081 | 0.005229 | 2.310384395 | 77911 |
| *CADM2* | rs1865251 | A | C | order | 7.27E-04 | -0.013002 | 0.003848 | -3.378898129 | 77731 |
| *CADM2* | rs1865251 | A | C | compliance | 3.82E-05 | 0.005946 | 0.001444 | 4.117728532 | 77731 |
| *CADM2* | rs1865251 | A | C | depression | 9.14E-02 | -0.004919 | 0.002915 | -1.687478559 | 77532 |
| *CADM2* | rs1865251 | A | C | assertiveness | 5.21E-01 | 0.004322 | 0.006727 | 0.642485506 | 77532 |
| *CADM2* | rs1865251 | A | C | altruism | 2.37E-02 | 0.006798 | 0.003006 | 2.261477046 | 77532 |
| *CADM2* | rs1865251 | A | C | neuroticism | 1.74E-05 | -0.052025 | 0.01211 | -4.296036334 | 77356 |
| *CADM2* | rs1865251 | A | C | ideas | 9.94E-01 | 0.000015 | 0.001884 | 0.007961783 | 77356 |
| *CADM2* | rs1865251 | A | C | extraversion | 1.48E-01 | 0.028084 | 0.019393 | 1.448151395 | 77356 |
| *CADM2* | rs1865251 | A | C | anxiety | 8.57E-06 | -0.01122 | 0.002521 | -4.450614835 | 77356 |
| *CADM2* | rs1865251 | A | C | self_discipline | 3.47E-05 | -0.015619 | 0.003772 | -4.140774125 | 77275 |
| *CADM2* | rs1865251 | A | C | openness | 9.42E-01 | 0.000287 | 0.003966 | 0.072365103 | 77275 |
| *CADM2* | rs1865251 | A | C | conscientiousness | 1.95E-05 | -0.013944 | 0.003266 | -4.269442743 | 77275 |
| *CADM2* | rs1865251 | A | C | agreeableness | 1.87E-04 | 0.056755 | 0.015193 | 3.735601922 | 77275 |
| *CADM2* | rs1865251 | A | C | aesthetics | 9.58E-01 | -0.00015 | 0.002851 | -0.052613118 | 77275 |
| *CADM2* | rs1865251 | A | C | risk_taking | 1.63E-05 | 0.018952 | 0.004397 | 4.310211508 | 140487 |
| *MSRA* | rs658385 | C | T | activity | 7.20E-04 | -0.017293 | 0.005114 | -3.38150176 | 77911 |
| *MSRA* | rs658385 | C | T | order | 7.68E-01 | 0.001109 | 0.003764 | 0.294633369 | 77731 |
| *MSRA* | rs658385 | C | T | compliance | 4.82E-04 | -0.004929 | 0.001412 | -3.490793201 | 77731 |
| *MSRA* | rs658385 | C | T | depression | 5.96E-07 | 0.014229 | 0.00285 | 4.992631579 | 77532 |
| *MSRA* | rs658385 | C | T | assertiveness | 6.25E-02 | -0.012253 | 0.006579 | -1.8624411 | 77532 |
| *MSRA* | rs658385 | C | T | altruism | 8.44E-01 | 0.000578 | 0.00294 | 0.196598639 | 77532 |
| *MSRA* | rs658385 | C | T | neuroticism | 1.11E-07 | 0.062873 | 0.011844 | 5.308426207 | 77356 |
| *MSRA* | rs658385 | C | T | ideas | 2.65E-02 | -0.004088 | 0.001842 | -2.219326819 | 77356 |
| *MSRA* | rs658385 | C | T | extraversion | 8.08E-03 | -0.050236 | 0.018968 | -2.648460565 | 77356 |
| *MSRA* | rs658385 | C | T | anxiety | 6.86E-05 | 0.009817 | 0.002466 | 3.980940795 | 77356 |
| *MSRA* | rs658385 | C | T | self_discipline | 9.64E-01 | 0.000168 | 0.00369 | 0.045528455 | 77275 |
| *MSRA* | rs658385 | C | T | openness | 9.82E-03 | -0.010014 | 0.003879 | -2.581593194 | 77275 |
| *MSRA* | rs658385 | C | T | conscientiousness | 9.41E-01 | -0.000237 | 0.003194 | -0.074201628 | 77275 |
| *MSRA* | rs658385 | C | T | agreeableness | 5.78E-02 | -0.028194 | 0.01486 | -1.89730821 | 77275 |
| *MSRA* | rs658385 | C | T | aesthetics | 2.12E-01 | -0.003478 | 0.002788 | -1.24748924 | 77275 |
| *MSRA* | rs658385 | C | T | risk_taking | 1.45E-02 | -0.010504 | 0.004299 | -2.443358921 | 140487 |
